# Industrial Applications of Different Parts of Flatland *Polygonum cuspidatum* by Combining Microwave-Assisted Extraction and Fermentation Process

**DOI:** 10.3390/plants14233572

**Published:** 2025-11-22

**Authors:** Chih-Yu Chen, Guey-Horng Wang, Jong-Tar Kuo, Pei-Ning Hsu, Yu-Chen Shen, Yen-Hsun Chen, Ying-Chien Chung

**Affiliations:** 1 Department of Tourism and Leisure, Hsing Wu University, New Taipei City 244012, Taiwan; chanos@ms25.hinet.net; 2 Research Center of Natural Cosmeceuticals Engineering, Xiamen Medical College, Xiamen 361008, China; 3 Department of Nutrition and Health Sciences, Kainan University, Taoyuan City 33857, Taiwan; 4 Department of Biological Science and Technology, China University of Science and Technology, Taipei City 115311, Taiwan

**Keywords:** antioxidant activity, cytotoxicity, fermentation, bioactivity

## Abstract

*Polygonum cuspidatum* is a well-known and versatile medicinal plant. In Taiwan, *P. cuspidatum* is typically found in central mountainous regions. Once acclimated, it can also thrive in flat areas, where it is known as flatland *P. cuspidatum*. Flatland *P. cuspidatum* has several advantages over alpine *P. cuspidatum*; for example, flatland *P. cuspidatum* grows faster and has larger leaves. This study enhanced the functionality of different parts of flatland *P. cuspidatum* (flowers, leaves, and rhizomes) by using microwave-assisted extraction (MAE) technology and Box–Behnken response surface methodology. Experiments revealed that the combination of MAE parameters that yielded optimal results was influenced by which plant part was used as input material. Regarding whitening activity, the extracts were ranked as follows: leaf > rhizome > flower. Leaf extracts had higher total flavonoid content, and rhizome extracts had higher total phenolic content. Regarding antiaging activity, the extracts were ranked as follows: rhizome > leaf > flower. The rankings for antimicrobial activity were as follows: leaf > rhizome > flower. Regarding anti-inflammatory activity, the extracts were ranked as follows: flower > rhizome > leaf. The rhizome extract exhibited slight cytotoxicity. UHPLC-UV-Q-TOF-HRMS/MS analysis identified 27, 34, and 37 bioactive compounds in the leaf, rhizome, and flower extracts, respectively. Given the relatively low pharmacological activity observed in the MAE-optimized flower extract, fermentation with *Aspergillus oryzae* was employed to enhance its efficacy. This process significantly enhanced the extract’s pharmacological properties, including its whitening, antiaging, and antimicrobial properties. Increased levels of 3-*O*-caffeoylquinic acid, decursin, quercetin, quercitrin, kaempferol, resveratrol, epicatechin gallate, resveratrol-3-*O*-D-(2-galloyl)-glucopyranoside, resveratrol-4′-*O*-β-D-glucoside, apigenin, emodin-8-*O*-(6′-*O*-malonyl)-glucoside, physcion, emodin, and torachrysone in the fermented flower extract likely contributed to its enhanced pharmacological activities. The results of this study indicate that the newly developed flatland *P. cuspidatum* extracts can be considered viable substitutes for alpine *P. cuspidatum* extracts.

## 1. Introduction

*Polygonum cuspidatum* Sieb. et Zucc., also known as Japanese Knotweed (*Reynoutria japonica*) or tiger stick, is an herbaceous perennial of the Polygonaceae family that is valued for its medicinal properties. The plant is native to East Asia and is widely distributed across Taiwan, China, South Korea, and Japan [1]. The plant is also widely distributed across North America, where it has become established as an invasive species. In Taiwan, *P. cuspidatum* is commonly found in the Central Mountain Range at elevations of 2000 to 3800 m, where the plant is typically referred to as alpine *P. cuspidatum*. In recent years, certain populations of this species have established themselves in flatland habitats at lower elevations. These populations are called flatland knotweed or flatland *P. cuspidatum*. Flatland *P. cuspidatum* has larger leaves, grows faster, and is easier to plant, transport, and process compared to alpine *P. cuspidatum*. If the pharmacological activity of flatland *P. cuspidatum* is comparable to that of alpine *P. cuspidatum*, this would be a positive attribute. Typically, medicinal and herbal extracts derived from *P. cuspidatum* are created using the plant’s roots and rhizomes [2]. Obtaining these parts requires one to uproot the entire plant, which presents a challenge with respect to the sustainable cultivation of *P. cuspidatum* for large-scale medicinal purposes [2]. Extracts can also be derived from the plant’s leaves and flowers. *P. caspidatum* has several medicinal effects. Actually, using aerial parts such as leaves and flowers represents a non-destructive harvesting strategy, making the flatland variant inherently more sustainable and scalable for industrial applications than traditional rhizome harvesting. The plant relieves cough, clears phlegm, enhances blood circulation, and is used to treat jaundice, ringworm, burns, joint pain, hyperlipidemia, and a range of inflammatory conditions [1]. The plant even has anticancer, antiviral, and cardiac protective effects [1,3,4]. Choi et al. (2020) produced an alpine *P. cuspidatum* rhizome extract by using a 50% ethanol solution and identified resveratrol, resveratrol glycoside, and emodin as its major constituents [5]. Yu et al. (2021) prepared alpine *P. cuspidatum* root and rhizome extracts by using a 95% ethanol solution, followed by purification through a macroporous resin process with sequential elution at 30%, 60%, and 95% ethanol concentrations [6]. They identified 31 bioactive compounds, including 12 anthraquinones, 7 diphenylethenes, 9 phenols, and 3 other compounds. The major constituents of those extracts were resveratrol, resveratrol glycoside, emodin, emodin-1-*O*-β-D-glucoside, and emodin-8-*O*-β-D-glucoside.

Since pharmacological activity varies depending on altitude and the plant part used, it is essential to evaluate the specific properties of the leaves, flowers, and rhizomes of flatland *P. cuspidatum*. Extraction methods and the part of the plant from which extracts are derived (flowers, leaves, stems, roots, and rhizomes) also influence the pharmacological activities of extracts [7]. More than 67 active constituents have been isolated from *P. cuspidatum* obtained from a variety of locations. The following are some of the isolated constituents and their effects: resveratrol (antiviral, anti-inflammatory, and antitumor effects), emodin (antibacterial, anti-inflammatory, whitening, and anticancer effects), rhein and emodin methyl ether (whitening effects), emodin-8-*O*-β-D-glucoside (antiviral effects), 6-hydroxyaloe emodin (citreorosein; anti-inflammatory and whitening effects), resveratrol glycoside (polydatin; anti-inflammatory, anticancer, and cardiovascular protective effects), catechins (antiviral effects), as well as stilbenes, coumarins, and anthraquinones, which exhibit a wide range of pharmacological activities [1,3,8,9].

Preliminary analyses of the active ingredients of extracts from different parts of *P. cuspidatum* have revealed distinct effects. Leaf extracts of the plant have antioxidant and anti-inflammatory effects; stem extracts have antioxidant effects; and rhizobium extracts have strong antioxidant, anti-inflammatory, and anticancer effects. Aqueous extracts of *P. cuspidatum* stems have been used to alleviate dry eye syndrome by reducing inflammation and apoptosis [10]. In addition, Xu et al. (2021) discovered that polydatin and resveratrol in *P. cuspidatum* extracts inhibited coronavirus replication, suggesting that the antibacterial and antiviral effects of *P. cuspidatum* extracts may have medical applications [11]. *P. cuspidatum* extracts may also have applications in the cosmeceutical industry. Quinty et al. (2022) extracted the upper parts (stems, branches, and leaves) and roots of *P. cuspidatum* by using 99.5% ethanol and ultrasound [12]. The extracts all had antiaging and whitening effects, and the root extract additionally had antioxidant effects. The findings of Quinty et al. (2022) indicate that the upper parts of *P. cuspidatum* have cosmeceutical applications [12]. Du et al. (2024) evaluated various commercially available *P. cuspidatum* extracts for tyrosinase inhibition in B16F10 cells and observed that a concentration of just 30 mg/L achieved 50–80% inhibition [9].

Bioactive compounds in *P. cuspidatum* are generally obtained using extraction and conversion technologies. Commonly used methods for obtaining such compounds include water distillation (stem extraction efficiency: 6.57%) [10], steam distillation, solvent extraction [4], ultrasound-assisted extraction (UAE) (root extraction efficiency: 12.4%) [13], supercritical fluid extraction (SFE) (rhizome extraction efficiency: 2.8%) [14], and microwave-assisted extraction (MAE) [15]. MAE is particularly suitable for extracting plant components from complex matrices. MAE is a simple and rapid technique that delivers high yields, requires only small amounts of solvent, and generates little waste; the technique also leads to minimal degradation of heat-sensitive compounds when temperature is properly regulated [16]. Consequently, MAE is widely employed to extract bioactive components from plants [17]. In the current study, in preliminary experiments, extracts were obtained from the flowers, leaves, and rhizomes of flatland and alpine *P. cuspidatum* by using 95% ethanol; the total phenolic content (TPC) in the flatland *P. cuspidatum* extracts was approximately 1.23–1.61 times higher than that in the alpine *P. cuspidatum* extracts, suggesting greater application potential for flatland *P. cuspidatum*. The operational parameters influencing the extraction efficiency of plant materials by MAE include extraction temperature, extraction time, liquid-to-solid (L/S) ratio, and extraction power [16]. Each of these parameters typically exhibits an optimal value for effective plant extraction. In general, elevated temperatures, extended extraction times, and increased extraction power enhance the extraction of bioactive ingredients from plant matrices. Nevertheless, excessively high levels of these conditions may result in the degradation of sensitive bioactive compounds [16]. Among these factors, extraction temperature is commonly regarded as the most critical variable. This study extracted bioactive compounds (by using MAE) from various parts of flatland *P. cuspidatum* and evaluated their tyrosinase inhibitory, antioxidant, antimicrobial, anti-inflammatory, and antiaging effects. Microorganisms produce greater quantities or higher concentrations of substances through fermentation. These substances are biologically active and enhance various biological functions. Enzymes produced during fermentation—such as xylanase, cellulase, and pectinase—can degrade plant cell structures, facilitating the release of bioactive compounds. Additionally, hydrolytic reactions like deglycosylation may alter the structure of certain metabolites, thereby affecting their interactions with reagents used in bioactivity assays. This study aimed to identify the bioactive compounds in the extracts and compare their activities. The physiological activity of biochemical products can be enhanced through fermentation, which modifies their naturally occurring molecular constituents. To further enhance the pharmacological properties of the flower extract obtained through MAE, fermentation was employed to enhance the bioactivity of the extracted compounds. Finally, the mechanisms underlying the physiological effects of the *P. cuspidatum* extracts were explored by analyzing the compositions and bioactive contents of the extracts.

## 2. Materials and Methods

### 2.1. Preparation of Raw Materials

The raw materials of *Polygonum cuspidatum* were collected from flatlands in Yunlin County, Taiwan. The collection was conducted after flowering, in August. The collected specimens were first dried in an oven at 50 °C for 48 h. The specimens were identified by Professor Bau-Yuan Hu from the China University of Science and Technology, Taiwan. They were then crushed and ground into powder using a pulverizer and passed through a 0.5 mm sieve. Finally, the powder was sealed and stored in a desiccator for later use.

### 2.2. MAE of P. cuspidatum

Despite the availability of several extraction techniques, including UAE and SFE, Microwave-Assisted Extraction (MAE) was selected as the most suitable technique for complex plant matrices due to its superior efficiency in recovering bioactive compounds. MAE conditions vary significantly depending on material being extracted. Preliminary experiments have established a feasible range of parameters. Based on these initial results, we defined the following single-factor ranges and evaluated antioxidant activities using total phenolic content (TPC) and total flavonoid content (TFC), expressed as absorbance, as indicators. In this study, a high-end microwave extractor (Milestone ETHOS X, Italy) operating at 350 W was used. The extracted materials included flatland *P. cuspidatum* flowers, leaves, and rhizomes. Extraction solvents comprised water, 95% ethanol, and ethyl acetate. The variables were set as follows:

(1) Extraction temperature (*x*_1_, °C)

The extraction time was set to 70 s, with a liquid-to-solid (L/S) ratio of 15 mL/g (e.g., 10 g of powder mixed with 150 mL of solvent), and the extraction temperature was 50–90 °C. The optimal extraction solvent was determined based on the extraction results.

(2) Extraction time (*x*_2_, s)

The extraction temperature was determined based on the results of (1). The L/S ratio was 15 mL/g, and the extraction time was 60–120 s.

(3) L/S (*x*_3_, mL/g)

The extraction temperature and time were based on the results of experiments (1) and (2), respectively, with a L/S ratio was 5–25 mL/g.

Based on the results above, the optimal extraction solvent (water, 95% ethanol, or ethyl acetate) for each part of flatland *P. cuspidatum* was selected. Response surface methodology (RSM) was then employed to optimize the extraction conditions using a Box–Behnken design (BBD). TPC and TFC were used as indicators of the antioxidant capacity of the extracts. According to BBD, 17 experimental runs were conducted, including 14 unique operating parameter combinations across three coded levels (−1, 0, 1) and 3 replicates at the center point (0, 0, 0). To model the relationships between the three parameters and TPC and TFC, a second-order polynomial equation was developed using Design-Expert software (version 12). Analysis of variance (ANOVA) was performed to assess the significance of each parameter’s effect on TPC and TFC, as well as the overall model fit (*F*-value and R^2^). Extracts obtained under the optimal conditions were freeze-dried for subsequent pharmacological activity analysis.

### 2.3. Fermentation of P. cuspidatum Flower Extract

Although the flower is the most sustainable plant part, *P. cuspidatum* flower extracts obtained through MAE exhibited the weakest pharmacological activity (whitening, anti-aging, antimicrobial). Therefore, fermentation is introduced as a necessary bioconversion strategy to enhance its efficacy and make it commercially viable in cosmeceutical applications. *Aspergillus oryzae* (BCRC 32288), obtained from the Bioresource Collection and Research Center (BCRC) in Taiwan and known for its use in fermentation of plant extracts [8], was used as the inoculum. This strain (*A. oryzae*) was cultured in potato dextrose broth (PDB) at 28 °C for 5 days. After incubation, submerged fermentation was conducted as follows: 1 mL of *A. oryzae* spore suspension (5 × 10^8^ spores/mL) was inoculated into 200 mL of sterile PDB containing 2 g of freeze-dried *P. cuspidatum* flower extract obtained through MAE. *A. oryzae* was also inoculated into sterile PDB without flower extract as a control. These mixtures were then incubated aerobically at 28 °C for 5 days. After fermentation, the fermentation broth was centrifuged at 8000× *g* for 10 min, and the supernatant was collected, filtered through a 0.45 mm filter, and concentrated using a rotary vacuum evaporator at 50 °C. The residues (fermented *P. cuspidatum* flower extract and fermented PDB) were subsequently freeze-dried and stored at −20 °C.

### 2.4. Measurement of Extracellular Tyrosinase Activity

The inhibition of mushroom tyrosinase activity serves as a screening tool for extracellular antityrosinase activity. The method is briefly described as follows: freeze-dried extracts of *P. cuspidatum* flowers, leaves, and rhizomes were dissolved in 1 mg/L DMSO solution and diluted to various concentrations. Then, 30 μL of this solution was sequentially mixed with 970 μL of phosphate-buffered saline (PBS) (0.05 mM, pH 6.8), 1 mL of tyrosine solution (100 mg/L), and 1 mL of tyrosinase solution (activity: 350 U/mL). After mixing for 20 min, the absorbance was measured at 490 nm by using an ultraviolet–visible (UV–vis) spectrophotometer (Thermo Fisher Scientific, Waltham, MA, USA) [18]. Furthermore, α-arbutin and kojic acid were used instead of *P. cuspidatum* extracts following a comparable protocol and served as positive controls. The half maximal inhibitory concentration (IC_50_) of the extract was defined as the concentration that inhibited 50% of the initial tyrosinase activity.

(1) $$\mathrm{Tyrosinase}\;\mathrm{inhibition}\;\left(\%\right)=\frac{\left[\left(A-B\right)-\left(C-D\right)\right]}{\left(A-B\right)}\times 100$$

A is the control group, representing the absorbance without extract. B is the absorbance of the blank group, which contains neither extract nor tyrosinase. C is the absorbance of the experimental group, containing both extract and tyrosinase. D is the absorbance of the blank group corresponding to C, which contains extract but no tyrosinase.

### 2.5. Measurement of Tyrosinase Activity and Melanin Content in Human HEMn Cells

This study directly investigated the whitening effects of extracts from the flower, leaf, and rhizome of flatland *P. cuspidatum* by assessing their ability to inhibit melanin production in human epidermal melanocytes (HEMn). Due to structural differences between human and mushroom tyrosinase, evaluating efficacy required more precise testing in human cells. HEMn cells (C-102-5C, Cascade Biologics, Inc., Portland, OR, USA) were cultured in Medium 254 (Cascade Biologics, Inc., Portland, OR, USA) and supplemented with a human melanocyte growth supplement (S-002-5, Cascade Biologics, Inc., Portland, OR, USA) to optimize the activity. During the culture period, 5% CO_2_ was continuously supplied, and the temperature was maintained at 37 °C. After 24 h of incubation, the cells were harvested and seeded into six-well culture plates at a density of 2 × 10^6^ cells per well. To test the whitening effects of the extracts, various concentrations were added and incubated for 24 h under the same culture conditions. Subsequently, the cells were prepared by rinsing with PBS, detaching them with trypsin, and lysing them via sonication. The active enzyme fraction was subsequently isolated in the supernatant through centrifugation. This supernatant was incubated with 2.5 mM L-DOPA in a 96-well microplate for 60 min. Tyrosinase activity in HEMn cells was assessed by monitoring the formation of dopachrome, achieved by recording the absorbance at 475 nm by using an Epoch ELISA reader (BioTek Instruments, Santa Clara, CA, USA) adapted from Wu et al. (2018) [19].

To measure the melanin content in HEMn cells, the cells were first washed with PBS and treated with trypsin as previously described. Subsequently, the cells were heated at 80 °C for 1 h in 10% DMSO prepared in 1 N NaOH. Following this treatment, the absorbance of the resulting solution was measured at 450 nm using an ELISA plate reader (Epoch, Biotek, Santa Clara, CA, USA). The absorbance readings were then compared to a standard curve constructed with synthetic melanin standards to determine the melanin content [19].

### 2.6. Assessment of TPC and TFC

TPC in the extract was determined using the Chen et al. method (2025) [20]. Briefly, 50 μL of Folin–Denis phenol reagent was added to 50 μL of the extract at varying concentrations and allowed to react for 3 min. Then, 50 μL of 10% Na_2_CO_3_ was added, and the mixture was incubated for an additional 60 min. The absorbance of the solution was measured at 760 nm in the solution was analyzed using an ELISA plate reader. For freeze-dried extracts, the absorbance values were converted to mg-gallic acid equivalent (GAE)/g-dried extract weight (DW) using a calibration curve. TFC was determined using the aluminum chloride colorimetric method [21]. Briefly, 0.6 mL of the extract at varying concentrations was mixed with 0.6 mL of 2% aluminum chloride and incubated for 60 min. The absorbance was then measured at 420 nm using a UV–vis spectrophotometer. For freeze-dried extracts, the absorbance values were converted to mg-rutin equivalent (RE)/g-DW using a calibration curve. The calibration curve for TPC was generated by using gallic acid, while the calibration curve for TFC was generated using rutin. Standard solutions were prepared by dissolving a precise amount of gallic acid or rutin powder in methanol, followed by serial dilution with the same solvent.

### 2.7. Assessment of DPPH and ABTS Free Radical Scavenging Activities

DPPH radical scavenging activity was determined based on Wu et al. (2018) [19]. Briefly, 50 μL of the extract was added to 100 μL of a 0.2 mM DPPH solution, mixed thoroughly, and then allowed to stand at room temperature for 10 min. The absorbance at 517 nm was then measured using an ELISA plate reader. Butylated hydroxytoluene (BHT) was used as positive control. DPPH radical scavenging activity was calculated using the following formula:

(2) $$\mathrm{DPPH}\;\mathrm{scavenging}\;\mathrm{activity}\;(\%)\;=\;\left\{1-\frac{\left(OD_{517}\;of\;sample\right)}{\left(OD_{517}\;of\;control\right)}\right\}\;\times 100$$

ABTS free radical scavenging activity was measured following the method adapted from Wu et al. (2018) [19]. Briefly, 7 mM ABTS was mixed with 2.45 mM ammonium persulfate and incubated in the dark at room temperature for 24 h. Then, 50 μL of the extract was combined with 100 μL of the ABTS solution and incubated at room temperature for 10 min. Absorbance at 734 nm was measured using an ELISA plate reader. BHT was used as a positive control. ABTS free radical scavenging activity was calculated using the following formula:

(3) $$\mathrm{ABTS}\;\mathrm{scavenging}\;\mathrm{activity}\;(\%)\;=\;\left\{1-\frac{\left(OD_{734}\;of\;sample\right)}{\left(OD_{734}\;of\;control\right)}\right\}\;\times 100$$

### 2.8. Measurement of Antiaging Activity

Matrix metalloproteinase-1 (MMP-1) is primarily responsible for the degradation of interstitial collagen in the skin. Inhibition of this enzyme can lead to antiaging effects. The assay method was adapted from Chen et al. (2022) [22] and is briefly described as follows: Human fibroblasts (CCD966SK) were obtained from the BCRC and seeded into 96-well microplates at approximately 2.0 × 10^4^ cells/well. The cells were cultured at 37 °C in a 5% CO_2_ atmosphere. After 24 h, the culture medium was removed and replaced with 200 μL of various concentrations of *P. cuspidatum* flower, leaf, or rhizome extracts, followed by incubation for 48 h. Subsequently, 100 μL of supernatant was collected and incubated with a human MMP-1 ELISA kit (RayBiotech, Norcross, GA, USA) for 2 h. After incubation, the MMP-1 detection antibody, streptavidin solution, and stop reagent were added sequentially according to the manufacturer’s instructions. The final reaction mixture was measured at 450 nm using an ELISA plate reader.

Elastase is primarily responsible for the breakdown of elastin in the skin. If the extract exhibits inhibitory activity against elastase, it could potentially achieve antiaging effects. The test method is briefly described as follows: 20 μL of various concentrations of *P. cuspidatum* extract from the flower, leaf, or rhizome was mixed with 50 μL of PBS (containing 100 mM HEPES, 500 mM NaCl, and 0.05% Tween 20) in a 96-well plate. Then, 50 μL of neutrophil elastase was added, and the mixture was incubated at 37 °C for 10 min. Finally, 5 μL of a 2 mM reaction substrate (MeOSuc-Ala-Ala-Pro-Val-pNA) was added and incubated for 5 min. The absorbance of the final solution was measured at 405 nm using an ELISA plate reader. A control group was performed using DMSO instead [19]. 1,10-Phenanthroline and gallic acid served as positive controls for elastase inhibition. The elastase inhibition efficiency of the extract was calculated as follows:

(4) $$\;\;\mathrm{Elastase}\;\mathrm{inhibition}\;\left(\%\right)=\left(1-\frac{OD_{405}\;of\;sample}{{\mathrm{OD}}_{405}\;\mathrm{of}\;\mathrm{control}\;}\right)\;\times 100\%$$

Collagenase is primarily responsible for collagen degradation in the skin. If the extract exhibits inhibitory activity against collagenase, antiaging effects may be achieved. Collagenase activity was measured using the fluorescent DQ-gelatin assay [23]. The method is briefly described as follows: First, 20 μL of various concentrations of *P. cuspidatum* extracts from flowers, leaves, or rhizomes were added to a 96-well plate. Then, 100 μL of collagenase (activity: 1 U/mL) and 15 μg/mL DQ gelatin were added, and the mixture was incubated for 15 min. Collagenase activity was then analyzed by measuring the fluorescence intensity at an excitation wavelength of 485 nm and an emission wavelength of 528 nm using a Synergy 2 microplate reader (BioTek Instruments, Santa Clara, CA, USA). Epigallocatechin-3-gallate (EGCG) was used as the positive control in the assays for inhibiting both MMP-1 and collagenase.

### 2.9. Measurement of Antimicrobial Activity

If the extracts from the flowers, leaves, or rhizomes of *P. cuspidatum* possess antimicrobial properties in addition to other pharmacological activities, this would increase the potential for use in industrial applications. In this study, the tested strains were selected based on the recommendations from the USP 51 antimicrobial effectiveness test, common skin pathogens, and prevalent drug-resistant bacteria [24]. The strains included seven bacterial species: Gram-positive (*Bacillus subtilis*, *Staphylococcus aureus*, *Enterococcus faecalis*, and *Cutibacterium acnes* and Gram-negative (*Escherichia coli*, *Pseudomonas aeruginosa*, and *Acinetobacter baumannii*); as well as three fungal species: *Candida albicans*, *Aspergillus brasiliensis*, and *Epidermophyton floccosum*. The antimicrobial effectiveness testing method is briefly described as follows: bacteria were cultured in Tryptic Soy Broth (TSB) at 32.5 °C, and fungi were cultured in Sabouraud Dextrose Broth (SDB) at 22.5 °C. When microbial proliferation reached approximately 10^6^ CFU/mL or 10^6^ spores/mL, 1 mL of the bacterial suspension or fungal spore suspension was mixed with 1 mL of extract at varying concentrations and 8 mL of freshly prepared TSB or SDB medium in a test tube. The minimum bactericidal concentrations (MBC) and minimum fungicidal concentrations (MFC) of the extracts were determined using a serial dilution method after 24 h of incubation for bacteria and 5 d for fungi.

### 2.10. Measurement of Anti-Inflammatory Activity

Inflammation is a common feature of many diseases; therefore, anti-inflammatory activity is widely applied in medicine, healthcare, and cosmeceuticals. During inflammation, the inflammatory mediators Interleukin-6 (IL-6) and Tumor necrosis factor-α (TNF-α) induce the expression of a complex set of genes in endothelial cells, indicating an inflammatory state [25]. Elevated nitric oxide (NO) levels are another marker of inflammation [26]. Consequently, this study used changes in IL-6, TNF-α, and NO levels as indicators of anti-inflammatory activity. The analytical method is briefly described as follows: experimental groups consisted of Raw264.7 cells (5 × 10^5^ cells/mL, obtained from the BCRC) cultured with 1 mg/L lipopolysaccharides (LPS) and varying concentrations of extracts from the flowers, leaves, or rhizomes of *P. cuspidatum*. Two control groups were included: one cultured with LPS but without any extract, and the other cultured with DMEM medium alone. To measure the expression levels (μg/L) of IL-6 and TNF-α, cells were mixed with LPS and then cultured with the extracts for 24 h. Subsequently, IL-6 and TNF-α levels were analyzed using the IL-6 Quantikine ELISA Kit and TNF-α Quantikine ELISA Kit, respectively (R&D Systems, Minneapolis, MN, USA). The NO content (μM) in cells was determined by mixing equal volumes of cell suspension with Griess reagent. After a 10 min reaction, the absorbance at 550 nm was measured using an ELISA plate reader. The amount of NO produced in the cells was estimated based on a standard curve established using NaNO_2_.

### 2.11. Assessment of Cell Survival and Wound Healing Ability

The significance of any pharmacological activity must be established under conditions that do not compromise cell survival. Therefore, assessing cytotoxicity or cell viability is essential. This study utilized four cell lines: HEMn (for melanin studies), CCD966SK (for antiaging activity and wound healing assays), Raw264.7 (for anti-inflammatory activity assays), and HaCaT (obtained from BCRC for wound healing assays). Cell viability assays were performed using the Cell Counting Kit-8 (CCK-8) (Dojindo, Kumamoto, Japan). Briefly, 10^4^ cells/well were seeded into a 96-well microplate, and extracts from the flowers, leaves, or rhizomes of flatland *P. cuspidatum* were then added at various concentrations for a 48 h incubation. Subsequently, 10 μL of CCK-8 solution was added to each well and incubated at 37 °C for 4 h. Absorbance at 450 nm was then measured using an ELISA plate reader. Cell viability was calculated using the following formula:

(5) $$\;\mathrm{Cell}\;\mathrm{viability}\;\left(\%\right)=\left(\frac{\;\;\;OD_{450}\;of\;sample}{OD_{450}\;of\;control}\right)\;\times 100\%\;\;\;\;$$

This study investigated the wound healing ability of extracts from the flowers of *P. cuspidatum*, using HaCaT and CCD-966SK cells as models for epidermal and dermal tissue repair, respectively. The evaluation followed the method recommended by Bazzicalupo et al. (2021) [27]. HaCaT and CCD-966SK cells were seeded in 6-well plates at a density of approximately10^4^ cells/well and cultured until a confluent monolayer formed. The cell layer was then carefully wounded twice using a sterile 200 μL plastic pipette tip. Various concentrations of the extract were added, and the cells were incubated for 24 h. After incubation, the cells were fixed with FineFIX and stained with toluidine blue O (TBO) solutions. Wound widths at 0 h and 24 h post-wounding were photographed using a stereomicroscope (Nikon SMZ445 MP, Japan) and measured with ImageJ software (version 1.51p).

### 2.12. Analysis of Chemical Composition

The chemical composition of extracts from the flowers, leaves, and rhizomes of flatland *P. cuspidatum* was analyzed using UPLC-PDA-QTOF/MS (Waters Acquity™ Ultra Performance LC system, USA) [6]. The separation column was a Waters CORTECS T3 column (2.1 × 100 mm, 1.6 μm). The flow rate was set at 0.40 mL/min, the column temperature was maintained at 35 °C, and the injection volume was 5 μL. The separation conditions were as follows: the mobile phase consisted of solvents A: 0.1% formic acid and B: acetonitrile. Qualitative analysis was performed using the following gradient: 1–40% B from 0 to 30 min, 40–99% B from 31 to 37 min, and 99% B from 37 to 40 min. Quantitative analysis was employed the following gradient: 1–15% B from 0 to 7 min, 15–40% B from 7 to 22 min, 40–95% B from 22 to 30 min, and 95–99% B from 30 to 32 min. The PDA detector had a detection range of 210–400 nm, with the detection wavelength set at 280 nm. TOF/MS conditions were as follows: capillary voltage, 2.3 kV; injection cone voltage, 40 V; gas temperature, 350 °C; gas flow rate, 600 L/h; extraction cone voltage, 4.0 V; cone gas flow rate, 40 L/h; source temperature, 100 °C; and scan time, 0.15 s. The scan range was 50–1200 *m/*z.

### 2.13. Statistical Analysis

Data were analyzed using one-way ANOVA followed by Duncan’s multiple range test and are presented as the means ± standard deviations of at least three replicates. Statistical analyses were performed using SPSS (version 20.0; IBM, Armonk, NY, USA). A *p*-value less than 0.05 was considered statistically significant.

## 3. Results and Discussion

### 3.1. MAE of Flatland P. cuspidatum—Single-Factor Experiments

The effects of extraction temperature, solvent choice, extraction time, and liquid-to-solid (L/S) ratio on the TPC andTFC of *P. cuspidatum* extracts were examined in single-factor experiments.

**Figure 1:**
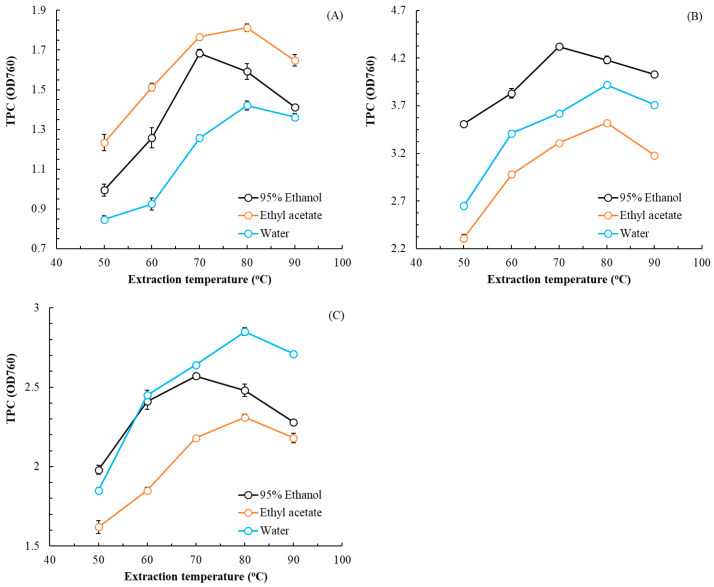
Effects of extraction temperature and solvent on TPC in flatland *P. cuspidatum* leaf (**A**), rhizome (**B**), and flower (**C**) extracts obtained using MAE (extraction conditions: extraction time, 70 s; L/S ratio, 15 mL/g; and microwave power, 350 W). Data are presented as the means and standard deviations of three independent experiments.

**Figure 2:**
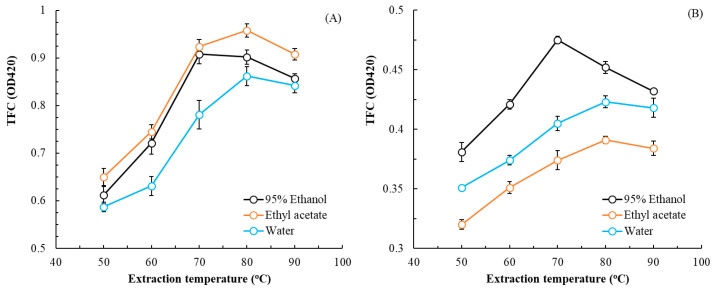

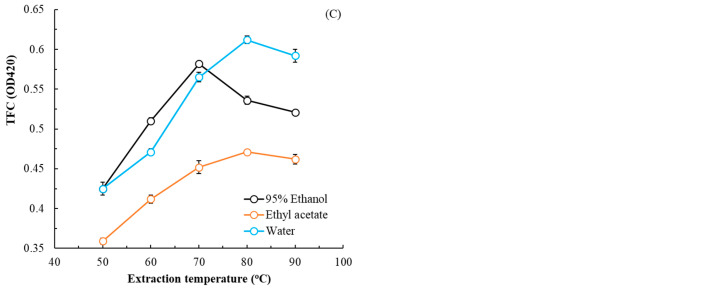
Effects of extraction temperature and solvent on TFC in flatland *P. cuspidatum* leaf (**A**), rhizome (**B**), and flower (**C**) extracts obtained using MAE (extraction conditions: extraction time, 70 s; L/S ratio, 15 mL/g; and microwave power, 350 W). Data are presented as the means and standard deviations of three independent experiments.

The effects of extraction temperature and solvent choice on TPC are illustrated in Figure 1, and the effects of these parameters on TFC are illustrated in Figure 2. For leaf, rhizome, and flower extracts, the optimal extraction temperatures were 80, 70, and 80 °C, respectively, and the optimal solvent choices were ethyl acetate, 95% ethanol, and water, respectively. As extraction temperature increased, TPC and TFC first increased and then decreased. The extract with the highest TFC was the leaf extract; the extract with the highest TPC was the rhizome extract. For extractions in subsequent experiments, these optimal solvent choices and temperatures were, respectively, applied for each part of the plant.

**Figure 3:**
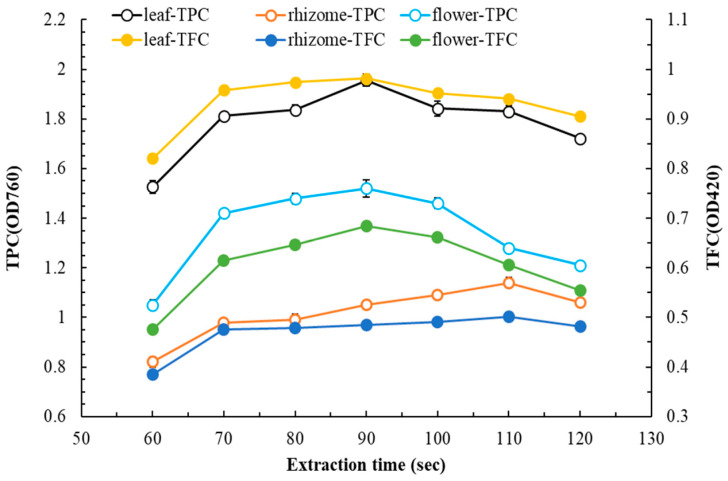
Effect of extraction time on TPC and TFC in flatland *P. cuspidatum* leaf, rhizome, and flower extracts obtained using MAE (extraction conditions: for leaf, extraction solvent, ethyl acetate; extraction temperature, 80 °C; L/S ratio, 15 mL/g; microwave power, 350 W; for rhizome, extraction solvent, 95% ethanol; extraction temperature, 70 °C; L/S ratio, 15 mL/g; microwave power, 350 W; for flower, extraction solvent, water; extraction temperature, 80 °C; L/S ratio, 15 mL/g; microwave power, 350 W). Data are presented as the means and standard deviations of three independent experiments.

Figure 3 illustrates how extraction time influences TPC and TFC. As extraction time increased, TPC and TFC first increased and then decreased. For leaf, rhizome, and flower extracts, the optimal extraction times were 90, 110, and 90 s, respectively (to facilitate comparison, TPC values for rhizome and flower extracts are presented at dilutions of ×4 and ×2, respectively). Extractions in subsequent experiments were completed using these optimal extraction times for each part of the plant.

**Figure 4:**
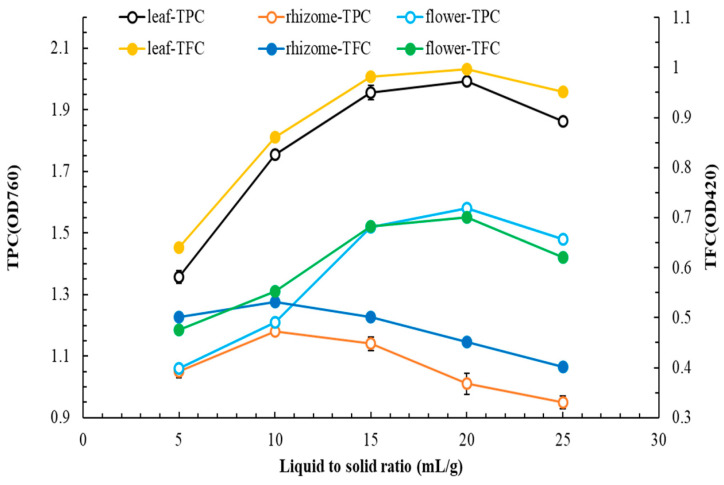
Effect of L/S ratio on TPC and TFC in flatland *P. cuspidatum* leaf, rhizome, and flower extracts obtained using MAE. (extraction conditions: for leaf, extraction solvent, ethyl acetate; extraction temperature, 80 °C; extraction time, 90 s; microwave power, 350 W; for rhizome, extraction solvent, 95% ethanol; extraction temperature, 70 °C; extraction time, 110 s; microwave power, 350 W; for flower, extraction solvent, water; extraction temperature, 80 °C; extraction time, 90 s; microwave power, 350 W). Data are presented as the means and standard deviations of three independent experiments.

Figure 4 illustrates how the L/S ratio influences TPC and TFC. As the L/S ratio increased, TPC and TFC first increased and then decreased. For leaf, rhizome, and flower extracts, the optimal L/S ratios were 20, 10, and 20, respectively.

### 3.2. MAE of Flatland P. cuspidatum—Experimental Design

We next optimized our MAE methodology by using a Box–Behnken design with three parameters (extraction temperature, extraction time, and L/S ratio). We generated 17 experimental sets and performed multiple regression analysis to establish correlations between the three parameters and TPC and TFC. The central coded values for extraction temperature, extraction time, and L/S ratio were 80 °C, 90 s, and 20, respectively, for leaf extracts; 70 °C, 110 s, and 10, respectively, for rhizome extracts; and 80 °C, 90 s, and 20, respectively, for flower extracts.

The leaf extract exhibited the highest TFC was the leaf extract, which serves as a key indicator of its quality. Similarly, the rhizome extract had the highest TPC, indicating the quality of the rhizome extract. The flower extract had a relatively high TFC, which serves as an indicator of its production. The relationship between extraction parameters and TFC in the leaf extract was as follows: *y* = 24.553 − 0.0768*x*_1_ − 0.009824*x*_2_ − 0.00823*x*_3_ − 0.000973*x*_1_^2^ − 0.000824*x*_2_^2^ − 0.00142*x*_3_^2^ − 0.000351*x*_1_*x*_2_ − 0.000613*x*_1_*x*_3_ − 0.0000514*x*_2_*x*_3_. Extraction temperature had a greater effect than extraction time and L/S ratio did (*p* < 0.05). The relationship between extraction parameters and TPC in the rhizome extract was as follows: *y* = −24.02 + 0.4563*x*_1_ − 0.2154*x*_2_ + 0.407*x*_3_ + 0.00589*x*_1_^2^ − 0.001026*x*_2_^2^ + 0.002455*x*_3_^2^ − 0.000258*x*_1_*x*_2_ + 0.00343 *x*_1_*x*_3_ − 0.00453 *x*_2_*x*_3_. Extraction temperature had a greater effect than extraction time and L/S ratio did, and L/S ratio had a greater effect than extraction time did (*p* < 0.05). The relationship between extraction parameters and TFC in the flower extract was as follows: *y* = −24.435 + 0.0901*x*_1_ + 0.00254*x*_2_ − 0.000957*x*_3_ + 0.000245*x*_1_^2^ + 0.000631*x*_2_^2^ − 0.00362*x*_3_^2^ + 0.000209*x*_1_*x*_2_ − 0.000938*x*_1_*x*_3_ − 0.0000862*x*_2_*x*_3_. Extraction temperature had a greater effect than extraction time and L/S ratio did (*p* < 0.05). We next performed analysis of variance. The *F*-value and R^2^ values were in the range of 2.53–6.74 and 0.9652–0.9874, respectively. These findings indicate a strong positive correlation between the experimental and predicted values, suggesting the suitability of this study’s models for value prediction. The extraction yields for rhizomes, leaves, and flowers were 13.6% ± 2.1%, 18.2% ± 3.5%, and 10.4% ± 1.6%, respectively. By comparison, hydrodistillation of stems yielded 6.57%, UAE of roots yielded 12.4%, and SFE of rhizomes yielded 2.8% [10,13,14]. Our analysis identified the following respective optimal MAE parameters for *P. cuspidatum* rhizomes, leaves, and flowers: extraction temperature, 72.6, 78.6, and 82.7 °C; extraction time, 108.7, 89.2, and 90.8 s; and L/S ratio, 11.2, 18.5, and 19.2. In the subsequent experiments in this study, we used freeze-dried extracts produced under these optimal conditions. These extracts derived from the rhizome, leaf, and flower are herein designated as MAE-optimized rhizome extract, MAE-optimized leaf extract, and MAE-optimized flower extract, respectively.

We determined the TPC and TFC of the extracts by using spectrophotometric assays, with absorbance measured at 760 nm for TPC and at 420 nm for TFC and with *y* = 0.00487*x* + 0.8713 (R^2^: 0.9992) for TPC and *y* = 0.00108*x* + 0.00917 (R^2^: 0.9992) for TFC. The TPC and TFC of the rhizome, leaf, and flower extracts were 395.10 mg-GAE/g-DW, 457.6 mg-RE/g-DW, and 320.66 mg-RE/g-DW, respectively.

### 3.3. Whitening and Antioxidant Effects of Flatland P. cuspidatum Extracts

Table 1 presents the extracellular and intracellular antityrosinase activity and melanin content of flatland *P. cuspidatum* extracts. The whitening activity of the leaf extract was greater than that of the rhizome extract, which in turn was greater than that of the flower extract. The leaf extract exhibited IC_50_ values of 58.4 ± 2.6, 66.8 ± 9.2, and 41.3 ± 5.8 mg/L for extracellular antityrosinase, intracellular antityrosinase, and intracellular antimelanogenic activities, respectively, with the intracellular antimelanogenic activities being the most pronounced. Intracellular whitening involves more complex mechanisms than extracellular whitening does and may entail upregulation of tyrosinase and tyrosinase-related proteins 1 and 2. As reported by Du et al. (2024) [9], extracts of *P. cuspidatum* showed whitening effects, although these were not significant. In our study, flatland *P. cuspidatum* extracts processed using MAE may yield higher quantities or concentrations of pharmacologically active ingredients.

The antioxidant properties of the flatland *P. cuspidatum* extracts of this study are summarized in Table 2. The leaf, rhizome, and flower extracts exhibited distinct antioxidant effects. The leaf extract had a higher TFC (460.4 ± 37.2 mg-RE/g-DW) than the rhizome and flower extracts did and had greater ABTS scavenging activity (IC_50_: 12.7 ± 3.9 mg/L). The rhizome extract had a higher TPC (396.3 ± 21.8 mg-GAE/g-DW) than the leaf and flower extracts did and had greater DPPH scavenging activity (IC_50_: 17.4 ± 2.3 mg/L). The leaf and flower extracts had similar TPCs but different antioxidant activities, possibly because of differences in the hydrophilic and hydrophobic compositions of the extracts. Notably, the antioxidant capacity of the rhizome extract was not inferior to that of commonly used commercial antioxidants (BHT). Choi et al. (2020) used 50% ethanol to extract material from the rhizomes of alpine *P. cuspidatum* [5]. Their extract had a TPC of 92.39 mg-GAE/g (less than that of the flatland *P. cuspidatum* extracts in the present study) and IC_50_ values of 105 and 45 mg/L for scavenging DPPH and ABTS radicals, respectively [5]. Alpine *P. cuspidatum* extract obtained using SFE exhibited IC_50_ values of 1800 and 87.5 mg/L for scavenging DPPH and ABTS radicals, respectively [14]. Notably, both of the aforementioned alpine extracts exhibited weaker effects than did all of the flatland *P. cuspidatum* extracts in the present study. The current results indicate that flatland *P. cuspidatum* may be an alternative to alpine *P. cuspidatum* in the cosmeceutical industry.

### 3.4. Antiaging and Antimicrobial Effects of Flatland P. cuspidatum Extract

Few studies have explored the antiaging effects of *P. cuspidatum* extract. The antiaging effects of this extract are influenced by several factors, including the part of the plant from which the extract is derived, the growing environment, and the extraction method. The antiaging activities (IC_50_) of the flatland *P. cuspidatum* extracts obtained in the current study are summarized in Table 3. The IC_50_ values for antiaging activity were 49.6–225.7, 63.2–276.5, and 95.6–263.1 mg/L for the leaf, rhizome, and flower extracts, respectively. Preliminary judgment based on individual indicators suggests that rhizome extracts have greater or equal antiaging effects relative to those of leaf extracts, which in turn have greater effects than flower extracts do. The current study’s rhizome extract had greater anti-MMP-1 and anticollagenase activities than the other two extracts did (IC_50_ values of 63.2 ± 7.5 and 201.4 ± 30.6 mg/L for anti-MMP-1 and anticollagenase activities, respectively). The leaf extract had the greatest antielastase activity, with an IC_50_ of 49.6 ± 3.7 mg/L. These results are comparable to those obtained for EGCG, 1,10-phenanthroline, and gallic acid. It was speculated that rhizome extract demonstrated the strongest antiaging effect compared to other plant parts. This demonstrates that selecting appropriate varieties and extraction technologies can enhance the pharmacological efficacy of plants. Furthermore, this study provides the first evidence that extracts of *P. cuspidatum* exhibit antiaging properties.

We explored bacterial resistance and related safety concerns by evaluating the antimicrobial activity of the flatland *P. cuspidatum* extracts. We examined resistance to *P. aeruginosa*, *A. baumannii*, *S. aureus*, *E. faecalis*, *B. subtilis*, *E. coli*, *C. albicans*, *A. brasiliensis*, *C. acnes*, and *E. floccosum*. MBCs and MFCs are listed in Table 4. The leaf extract had the strongest antimicrobial activity, and the flower extract had the weakest. The leaf extract had MBC and MFC values of 150–400 and 300–350 mg/L, respectively, demonstrating good antimicrobial efficacy. The leaf extract having greater antimicrobial activity than the other extracts may be related to its relatively higher TPC. The rhizome extract’s antibacterial effects were stronger for Gram-positive bacteria (MBC: 300–400 mg/L) than for Gram-negative bacteria (MBC: 517–1700 mg/L), possibly because of the different antibacterial mechanisms of its active ingredients. Li et al. (2016) demonstrated that emodin, a compound found in the *P. cuspidatum* rhizome extract, effectively inhibits the growth of *Haemophilus parasuis* (MBC: 64 mg/L) [28]. Emodin is a major component of *P. cuspidatum* leaf extract (Table 5) and may be one of the extract’s key antibacterial chemicals. The flower extract had only weak antimicrobial activity, possibly because it lacked key antimicrobial compounds. In the upcoming experiments, we will explore how to enhance the concentration of active compounds in the MAE-optimized flower extract through the fermenta.

### 3.5. Anti-Inflammatory Effects, Wound Healing Ability, and Cytotoxicity of Flatland P. cuspidatum Extracts

**Figure 5:**
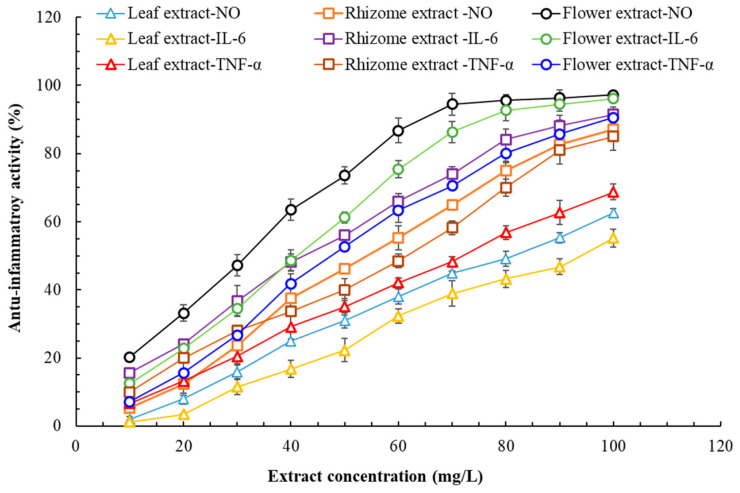
Anti-inflammatory activity of flatland *P. cuspidatum* leaf, rhizome, and flower extracts obtained using MAE under optimal conditions. Data are presented as the means and standard deviations of three independent experiments.

The anti-inflammatory activity of the *P. cuspidatum* extracts of this study is presented in Figure 5. The flower extract—followed by the rhizome and leaf extracts—was the most effective at inhibiting NO production and TNF-α activity. Regarding the inhibition of IL-6 activity, the effectiveness ranked as follows: the flower extract was as effective as or more effective than the rhizome extract, which was more effective than the leaf extract. Overall, the flower extract had the greatest anti-inflammatory activity, the rhizome extract had the second-greatest anti-inflammatory activity, and the leaf extract had the weakest anti-inflammatory activity. Regarding the flower extract’s anti-inflammatory activities, we obtained IC_50_ values ranging from 32.6 ± 0.9 to 48.3 ± 1.2 mg/L. In previous research, Ke et al. (2023) observed reduced concentrations of pro-inflammatory proteins in samples treated with *P. cuspidatum* rhizome extract [2]. According to Chen et al. (2021), the anti-inflammatory effects of *P*. *cuspidatum* are related to polydatin [29]. Accordingly, the flower extract having greater anti-inflammatory activity than the other extracts may be related to its relatively higher polydatin content (Table 5). Hadzik et al. (2023) demonstrated that alpine *P. cuspidatum* rhizome extract promotes wound healing and that 2000 mg/L of rhizome extract promoted the synthesis of type III collagen in HGF cells [30]. Given that *P. cuspidatum* flower extract has strong antioxidant capacity and excellent anti-inflammatory activity, it likely also promotes wound healing. Accordingly, we evaluated the wound healing ability of *P. cuspidatum* flower extract.

**Figure 6:**
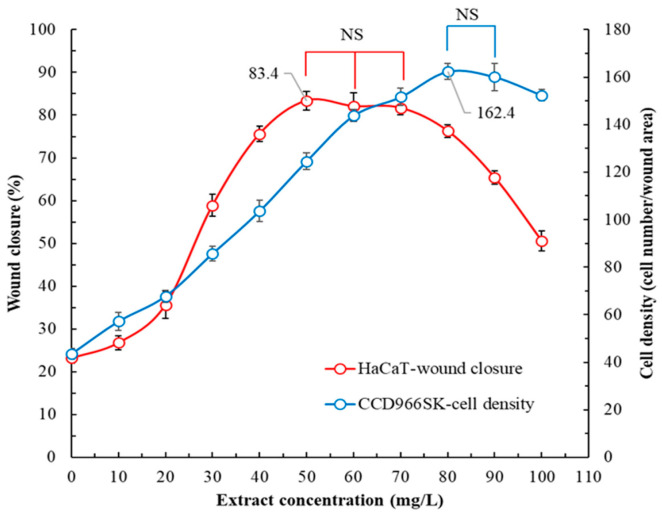
Wound healing abilities of flatland *P. cuspidatum* flower extracts obtained using MAE under optimal conditions. Data are presented as the means and standard deviations of 50 wounded cells. (NS, nonsignificant; *p* > 0.05).

The wound healing ability of the flatland *P. cuspidatum* flower extract is illustrated in Figure 6. The optimal concentration of flower extract for effective wound healing differed with cell type in our evaluation of epidermal cells (the HaCaT cell line) and dermal cells (the CCD966SK cell line). The optimal concentration for epidermal cells was 50 mg/L. This concentration resulted in a wound healing rate of 83.4% ± 2.2%. The optimal concentration for dermal cells was 80 mg/L, with it yielding a cell density value of 162.4 ± 3.5 cells/wound area. The wound healing ate in a control group (not treated with the flower extract) was 23.1% ± 1.2%, with a cell density value of 43.5 ± 2.3 cells/wound area. These results indicate that treatment with the extract can increase wound healing ability by approximately 3.73-fold. Wu et al. (2012) reported that administering a 10% *P. cuspidatum* rhizome extract to injured mice increased wound healing ability by 1.43-fold [31].

**Figure 7:**
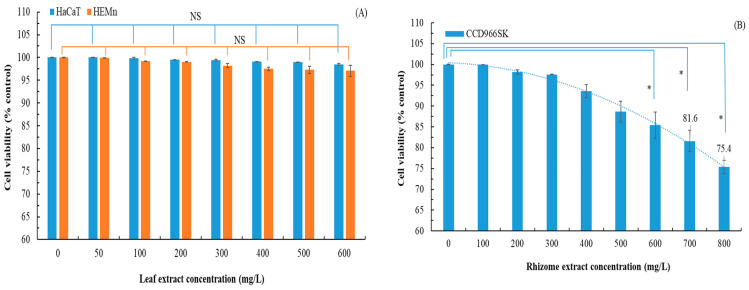

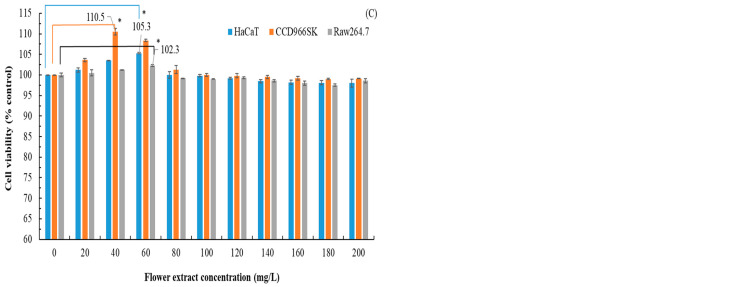
Cytotoxic effects of flatland *P. cuspidatum* leaf (**A**), rhizome (**B**), and flower (**C**) extracts obtained using MAE under optimal conditions. Data are presented as the means and standard deviations of three independent experiments. (NS, nonsignificant; *p* > 0.05, significance, * *p* < 0.05).

*P. cuspidatum* rhizome extract was not cytotoxic to HGF cells at concentrations up to 2000 mg/L [30]. According to Du et al. (2024), *P. cuspidatum* rhizome extract is mildly cytotoxic to B16F10 cells, with a 60% survival rate observed at 62.5 mg/L [9]. In this study, we explored whether *P. cuspidatum* leaf extract was cytotoxic to HaCaT and HEMn cells. On the basis of IC_50_ values obtained from previous experiments, we used a test concentration of 600 mg/L or less. We also explored whether *P. cuspidatum* rhizome extract was cytotoxic to CCD966SK cells, using a test concentration of 800 mg/L or less. We also explored whether *P. cuspidatum* flower extract was cytotoxic to HaCaT, CCD966SK, and Raw264.7 cells, using a test concentration of 200 mg/L or less. Figure 7 illustrates the cytotoxic effects of the extracts from flatland *P. cuspidatum*. The leaf extract was not cytotoxic; cell viability was 97.1% ± 1.2% at 600 mg/L. By contrast, the rhizome extract exhibited pronounced cytotoxicity, with cell viability dropping below 80% (the established cytotoxicity threshold) at 800 mg/L [32]. The flower extract was not cytotoxic; cell viability was 98.0% ± 1.0% at 200 mg/L. At lower concentrations, the flower extract even promoted cell growth. Therefore, flatland *P. cuspidatum* extracts obtained via MAE are biosafe across a broad range of concentrations. Directly contrast the high pharmacological potential of the rhizome with its cytotoxicity limitations. This is particularly important because the flower extract was not only safe but also promoted cell proliferation at lower concentrations. Addressing this trade-off would offer a more balanced and critical evaluation of each plant part’s potential.

### 3.6. Effects of Fermentation on Pharmacological Activities of P. cuspidatum Flower Extract

**Figure 8:**
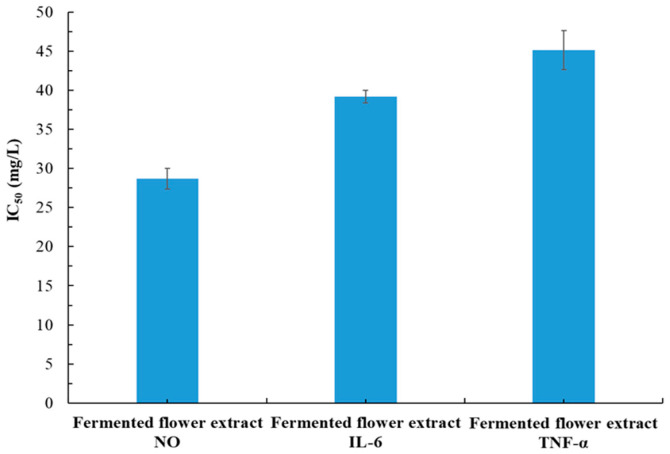
IC_50_ value of fermented flatland *P. cuspidatum* flower extract for its anti-inflammatory effect. Data are presented as the means and standard deviations of three independent experiments. This extract was produced using MAE under optimal conditions followed by fermentation with *A. oryzae* for 5 days.

Among the three MAE-optimized extracts analyzed in this study, the flower extract had the weakest whitening, antiaging, and antibacterial effects. We explored whether fermentation would enhance the pharmacological effects of the MAE-optimized flower extract. The IC_50_ value for the inhibition of intracellular melanin by the fermented flower extract was 57.9 ± 7.1 mg/L (Table 1). The IC_50_ values for the extract’s antiaging activities (against MMP-1, collagenase, and elastase) were 54.6 ± 7.1, 200.8 ± 27.3, and 55.2 ± 6.3 mg/L, respectively (Table 3). The MBC and MFC values were 200–400 and 350–500 mg/L, respectively (Table 4). The IC_50_ values for the extract’s anti-inflammatory activities ranged from 28.7 ± 1.3 to 45.1 ± 2.5 mg/L (Figure 8). Overall, fermentation increased the whitening effect by 7.52-fold, the antiaging effect by 1.31- to 1.86-fold, and the antimicrobial effect by 1.88- to 4.29-fold and had no obvious effect on the extract’s anti-inflammatory properties, wound healing abilities, or cytotoxicity. The increased whitening, antiaging, and antimicrobial effects were likely because of considerably increases in polyphenol concentrations, with TPC increasing 3.52-fold (from 102.4 ± 10.1 to 360.5 ± 19.2 mg-GAE/g-DW) and TFC increasing 1.38-fold (from 323.5 ± 26.7 to 446.4 ± 30.6 mg-RE/g-DW; Table 2). The concentrations of the active ingredients in the fermented extract are detailed in Table 5. In this study, the fermentation of MAE extracts by *A. oryzae* resulted in elevated concentrations of phenolic and flavonoid compounds, alongside the biosynthesis of new phenolic constituents, which collectively contributed to a marked enhancement of diverse physiological properties. These results are consistent with the findings reported by Wu et al. (2018) [19], who employed the same fungal genus, *A. niger* and demonstrated that fermentation enhanced the antioxidant activity of *Magnolia officinalis* extracts. Additionally, He et al. (2024) observed that fermentation of *P. cuspidatum* roots with *Penicillium rubens* resulted in a 9.18-fold increase in resveratrol yield relative to the unfermented extract [33]. Nonetheless, the precise mechanisms driving these bioactivities have yet to be elucidated.

### 3.7. Major Chemical Compositions and Contents of P. cuspidatum Extracts

The major chemical compositions and their relative contents in the *P. cuspidatum* leaf, rhizome, and flower extracts are listed in Table 5. Chemical species with relative concentrations of <0.1% were excluded. The leaf, rhizome, flower, and fermented flower extracts had 27, 34, 37, and 38 bioactive compounds, respectively. The leaf extract was dominated by flavonoids, anthraquinones, and stilbenes, which accounted for 37.78%, 28.60%, and 26.63% of the total content, respectively. Resveratrol was the most abundant compound, followed by emodin, quercitrin, emodin-3-methyl ether, polydatin, 5-*O*-caffeoylquinic acid, citreorosein, and quercetin 3-*O*-pentoside. The rhizome extract primarily consisted of anthraquinones, stilbenes, and naphthols, which accounted for 56.19%, 21.09%, and 13.41% of the total content, respectively. Emodin was the most abundant compound, followed by emodin-8-*O*-β-D-glucoside. Other notable compounds included resveratrol, torachrysone-8-*O*-glucoside, emodin-1-*O*-β-D-glucoside, and torachrysone. The flower extract primarily consisted of anthraquinones, flavonoids, and stilbenes, which accounted for 36.02%, 29.98%, and 29.09% of the total content, respectively. Citreorosein was the most abundant compound, followed by resveratrol. Other notable compounds were polydatin, kaempferol, quercetin, quercetin 3-*O*-pentoside, emodin, and quercitrin. The fermented flower extract was similarly dominated by anthraquinones, flavonoids, and stilbenes, which accounted for 33.38%, 30.52%, and 29.63% of the total content, respectively. The relative dominance of these compounds was largely unaffected by fermentation, with the exception of epicatechin gallate, which was not present before fermentation. The relative contents of resveratrol, kaempferol, quercetin, emodin, quercitrin, 3-*O*-caffeoylquinic acid, decursin, resveratrol-3-*O*-D-(2-galloyl)-glucopyranoside, resveratrol-4′-*O*-β-D-glucoside, apigenin, emodin-8-*O*-(6′-*O*-malonyl)-glucoside, physcion, and torachrysone increased. Increases in the relative contents of these compounds may enhance the pharmacological activity of the flower extract.

Choi et al. (2020) produced a rhizome extract by using a 50% ethanol and identified resveratrol, polydatin, and emodin as its major constituents [5]. Yu et al. (2021) employed a 95% ethanol and identified resveratrol, polydatin, emodin, physcion, emodin-1-*O*-β-D-glucoside, and emodin-8-*O*-β-D-glucoside as major constituents [6]. Quinty et al. (2022) performed UAE with a 99.5% ethanol and identified resveratrol, polydatin, and emodin as the major constituents [12]. These findings are consistent with the major compounds identified in the MAE-optimized rhizome extract, except for polydatin, physcion, and emodin-1-*O*-β-D-glucoside, which were not predominant. Quinty et al. (2022) produced extracts by using aerial parts of *P. caspidatum* (i.e., the stems, branches, and leaves) [12]. They performed UAE with a 99.5% ethanol and identified chlorogenic acid, catechin, polydatin, resveratrol, emodin, quercitrin, and torachrysone glucoside as the major constituents, which match those identified in the MAE-optimized leaf extract, although the current study did not detect chlorogenic acid or torachrysone glucoside.

The whitening activity of the leaf extract may be attributable to resveratrol, 3-*O*-caffeoylquinic acid, 5-*O*-caffeoylquinic acid, catechin, epicatechin, and apigenin [34,35,36,37]. This extract’s antimicrobial activity is likely caused by the presence of 3-*O*-caffeoylquinic acid, *p*-coumaroylquinic acid, quercitrin, and emodin-3-methyl ether [38,39,40,41]. The antiaging activity of the rhizome extract may be attributable to resveratrol, epicatechin gallate, and emodin-8-*O*-(6′-*O*-malonyl)-glucoside [42,43,44]. Additionally, the anti-inflammatory ability of the flower extract may be attributable to galloyl glucoside, resveratroloside, polydatin, quercetin, quercetin 3-*O*-pentoside, kaempferol, citreorosein, and physcion [45,46,47,48,49,50,51]. Fermentation increased the whitening activity of the MAE-optimized flower extract. This increase is primarily attributable to increased levels of decursin (4.2-fold), resveratrol-4′-*O*-β-D-glucoside (1.4-fold), and apigenin (1.5-fold) [36,52,53]. Fermentation also increased the MAE-optimized flower extract’s antiaging activity. This increase was mainly related to a 5.2-fold increase in 3-*O*-caffeoylquinic acid, the emergence of epicatechin gallate as a new product, and a 1.7-fold increase in emodin-8-*O*-(6′-*O*-malonyl)-glucoside [42,44,54]. Fermentation also increased the antimicrobial activity of the MAE-optimized flower extract. This increase is primarily attributable to increases in resveratrol-3-*O*-D-(2-galloyl)-glucopyranoside (1.4-fold), physcion (1.6-fold), and torachrysone (1.8-fold) [55,56,57]. The mechanisms underlying these activities remain unknown. Further validation through molecular docking studies and testing with purified compounds is required. It is proposed that the microbes employ enzymes that directly modify the plant’s compounds through the following: (1) deglycosylation, identified as the most prominent modification, frequently leading to an elevated concentration of aglycones [33]; (2) hydrolysis and degradation, whereby microorganisms decompose complex molecular structures [58]; and (3) biosynthesis of novel metabolites, wherein the fermenting microbes may generate new bioactive compounds [59]. Nonetheless, the exact biochemical pathways underlying these fermentation processes remain to be fully elucidated and warrant further investigation.

## 4. Conclusions

To the best of our knowledge, this study is the first to elucidate the various pharmacological activities of flatland *P. cuspidatum* extracts produced using MAE alone or in combination with fermentation. Notably, our fermentation process significantly enhanced the pharmacological (i.e., whitening, antiaging, and antimicrobial) activities of the MAE-optimized flower extract. The phenolic and flavonoid compounds in the flower extract underwent biosynthesis and biotransformation during fermentation. These processes increased the extract’s TPC and TFC, thereby enhancing the physiological activity of the extract. Additionally, the extracts exhibited no significant cytotoxic effects on various tested cell lines within medicinal concentration ranges. In conclusion, *P*. *cuspidatum* leaf extracts have potential for use in whitening and antimicrobial products; rhizome extracts have potential for use in antiaging products; flower extracts have potential for use in anti-inflammatory products; and fermented flower extracts have broad potential for use in multiple products. Based on the data presented, the flatland extract is not only a viable substitute but potentially superior to the alpine variety. Further research is necessary to determine the suitability of flatland *P*. *cuspidatum* extract for clinical and pharmaceutical use. In summary, flatland *P. cuspidatum* extracts could replace alpine *P. cuspidatum* extracts for medicinal purposes.

## Figures and Tables

**Table 1:** Whitening properties of flatland *P. cuspidatum* extracts (IC_50_, mg/L).

	In Vitro Antityrosinase Activity	In Vivo Antityrosinase Activity	Melanin Content in HEMn Cells
Leaf extract	58.4 ± 2.6	66.8 ± 9.2	41.3 ± 5.8
Rhizome extract	72.6 ± 11.7	81.5 ± 15.5	80.7 ± 26.8
Flower extract	417.1 ± 36.5	436.7 ± 41.3	435.1 ± 34.7
Fermented flower extract	−	−	57.9 ± 7.1

The fermented flower extract was produced using MAE under optimal conditions followed by fermentation with *A. oryzae* for 5 days. α-Arbutin and kojic acid were used as positive controls for in vitro antityrosinase activity, with IC_50_ values of 124.5 ± 8.6 mg/L and 12.4 ± 0.8 mg/L, respectively.

**Table 2:** Antioxidant properties of flatland *P. cuspidatum* extracts.

	DPPH (IC_50_, mg/L)	ABTS (IC_50_, mg/L)	TPC (mg-GAE/g-DW)	TFC (mg-RE/g-DW)
Leaf extract	62.7 ± 9.2	12.7 ± 3.9	127.5 ± 14.6	460.4 ± 37.2
Rhizome extract	17.4 ± 2.3	40.6 ± 5.6	396.3 ± 21.8	255.5 ± 19.4
Flower extract	105.2 ± 5.8	18.5 ± 2.3	102.4 ± 10.1	323.5 ± 26.7
Fermented flower extract	−	−	360.5 ± 19.2	446.4 ± 30.6

The fermented flower extract was produced using MAE under optimal conditions followed by fermentation with *A. oryzae* for 5 days. BHT was used as a positive control in the DPPH scavenging assay with an IC_50_ value of 51.6 ± 2.7 mg/L, and in the ABTS scavenging assay with an IC_50_ value of 43.2 ± 3.8 mg/L.

**Table 3:** Antiaging activity of flatland *P. cuspidatum* (IC_50_, mg/L).

	MMP-1	Collagenase	Elastase
Leaf extract	92.1 ± 10.4	225.7 ± 23.4	49.6 ± 3.7
Rhizome extract	63.2 ± 7.5	201.4 ± 30.6	276.5 ± 12.5
Flower extract	95.6 ± 8.2	263.1 ± 14.3	102.6 ± 15.4
Fermented flower extract	54.6 ± 7.2	200.8 ± 27.3	55.2 ± 6.3

The fermented flower extract was produced using MAE under optimal conditions followed by fermentation with *A. oryzae* for 5 days. EGCG was used as a positive control in the MMP-1 assay, with an IC_50_ value of 91.2 ± 8.7mg/L, and in the collagenase assay, with an IC_50_ value of 65.7 ± 4.6 mg/L. In the elastase assay, 1,10-phenanthroline (IC_50_: 47.2 ± 5.1 mg/L) and gallic acid (IC_50_: 128.7 ± 11.3 mg/L) were used as positive controls.

**Table 4:** MBC and MFC of flatland *P. cuspidatum* extracts.

		Leaf Extract	Rhizome Extract	Flower Extract	Fermented Flower Extract
*Bacteria* (MBC, mg/L)					
	*B. subtilis*	200 ± 35.4	300 ± 35.4	800 ± 70.7	400 ± 0.0
G(+)	*S. aureus*	150 ± 35.4	367 ± 20.4	700 ± 35.4	200 ± 0.0
	*E. faecalis*	200 ± 0.0	400 ± 0.0	750 ± 35.4	400 ± 0.0
	*C. acnes*	233 ± 20.4	300 ± 35.4	1000 ± 70.7	367 ± 20.4
	*E. coli*	200 ± 0.0	517 ± 54.0	450 ± 35.4	200 ± 0.0
G(−)	*P. aeruginosa*	217 ± 20.4	1700 ± 141.4	450 ± 0.0	200 ± 0.0
	*A. baumannii*	400 ± 35.4	1500 ± 0.0	500 ± 0.0	217 ± 20.4
*Fungi* (MFC, mg/L)					
*C. albicans*		350 ± 0.0	500 ± 70.7	1500 ± 70.7	350 ± 35.4
*A. brasiliensis*		300 ± 0.0	833 ± 73.6	– *	500 ± 70.7
*E. floccosum*		350 ± 35.4	1000 ± 0.0	–	500 ± 70.7

The fermented flower extract was produced using MAE under optimal conditions followed by fermentation with *A. oryzae* for 5 days. * >2000 mg/L.

**Table 5:** Major chemical compositions and relative contents of flatland *P. cuspidatum* extracts.

Chemical Compounds	RT (min)	Leaf Extract	Rhizome Extract	Flower Extract	Fermented Flower Extract
Relative Content (%)					
Galloyl glucoside	3.41	0	0.13	0.23	0.25
3-*O*-Caffeoylquinic acid	3.85	3.12	0	0.15	0.78
*p*-coumaroylquinic acid	4.32	2.89	0	0.21	0.26
5-*O*-caffeoylquinic acid	4.56	5.41	1.45	2.14	2.25
Decursin	5.81	0.57	3.82	0.25	1.05
Catechin-5-*O*-glucoside	7.12	2.28	0.12	1.71	1.02
Catechin	8.13	3.45	0.31	1.23	1.03
Epicatechin	10.72	3.15	0.33	1.62	1.14
Resveratroloside	11.57	0.57	1.74	2.14	0.95
Polydatin	11.83	7.32	0.48	10.62	10.37
Polydatin gallate	12.05	0.18	0.62	0.53	0.48
Quercetin	12.27	2.34	0	6.12	7.35
Quercetin 3-*O*-pentoside	12.54	5.12	0	5.63	4.86
Quercitrin	13.71	10.34	2.87	4.68	5.27
Kaempferol	13.85	3.65	0	8.57	9.24
Resveratrol	14.52	17.62	13.63	12.74	13.68
Epicatechin gallate	15.87	0.23	0.63	0	0.35
Resveratrol-3-*O*-D-(2-galloyl)-glucopyranoside	16.48	0	3.21	2.08	2.94
Resveratrol-4′-*O*-β-D-glucoside	17.26	0	0.12	0.71	0.48
Emodin-1-*O*-β-D-glucoside	18.52	0.35	7.72	0.92	0.71
Resveratrol-4′-*O*-β-D-glucoside (isomer)	19.08	0.71	0.66	0.27	0.38
Apigenin	19.35	2.45	0.28	0.42	0.61
Luteoin-7-*O*-glucoside	20.01	0	0.24	0.31	0.22
Emodin-*O*-(galloyl)-glucoside	20.28	0	0.29	1.92	1.51
Torachrysone-8-*O*-glucoside	21.37	0	8.23	0.83	0.62
Emodin-8-*O*-β-D-glucoside	21.68	1.12	18.11	2.72	2.09
Emodin-*O*-(galloyl)-glucoside	23.14	0	0.18	1.07	0.84
Acetylemodin-*O*-glucoside	24.18	0	0.84	0	0
Emodin-8-*O*-(6′-*O*-malonyl)-glucoside	23.57	0.95	0.83	0.43	0.72
Emodin-6-*O*-glucoside	24.62	0.32	0.24	0.51	0.38
Emodin-1-questin	24.81	0.27	1.54	0.36	0.13
Hydroxyl aloe-emodin	24.99	0	0.18	0.24	0.15
Torachrysone-8-*O*-(acetyl)-glucoside	25.34	0	0.42	0.57	0.31
citreorosein	25.92	5.17	1.06	16.61	15.39
Physcion glucoside	26.14	0	0.52	2.61	2.04
Physcion	27.35	0.27	0.37	1.24	1.93
emodin-3-methyl ether	28.71	7.91	1.52	1.67	1.24
Emodin	30.67	12.24	22.55	5.41	6.03
Torachrysone	31.51	0	4.76	0.53	0.95

The fermented flower extract was produced using MAE under optimal conditions followed by fermentation with *A. oryzae* for 5 days. RT indicates retention time.

## Data Availability

All data generated or analyzed during this study are included in this published article.
